# Supplementary material for: Causal interplay between lactose intolerance and gut microbiota: a combined bidirectional Mendelian randomization and in vivo validation study
Source: Front Nutr. 2026 Jun 1;13:1803337. doi: 10.3389/fnut.2026.1803337 (PMC13265576; doi:10.3389/fnut.2026.1803337)
Supplement: Supplementary file 1 [file Data_Sheet_1.zip › supplementary materials/Forward/forest plot/ebi-a-GCST90027778.finngen_R12_E4_LACTONAS.pdf]

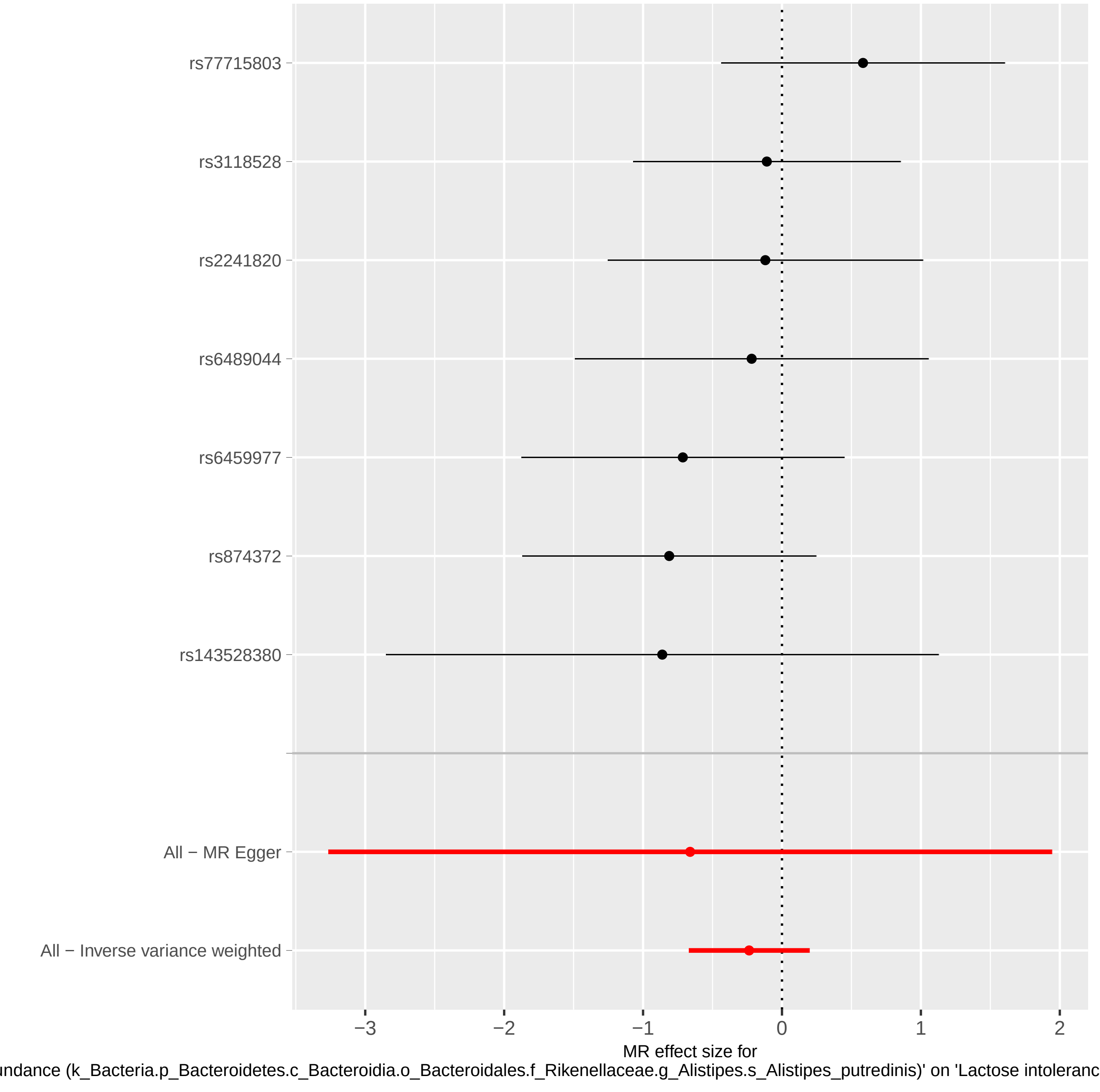

rs77715803
rs3118528
rs2241820
rs6489044
rs6459977
rs874372
rs143528380
All − MR Egger
All − Inverse variance weighted
−3
−2
−1
0
1
2
MR effect size for
undance (k_Bacteria.p_Bacteroidetes.c_Bacteroidia.o_Bacteroidales.f_Rikenellaceae.g_Alistipes.s_Alistipes_putredinis)' on 'Lactose intoleranc
